# Draft Genome Sequence of *Bacillus amyloliquefaciens* EBL11, a New Strain of Plant Growth-Promoting Bacterium Isolated from Rice Rhizosphere

**DOI:** 10.1128/genomeA.00732-14

**Published:** 2014-07-24

**Authors:** Yinghuan Wang, Xiaoyi Wang, Paul Greenfield, Decai Jin, Zhihui Bai

**Affiliations:** a CSIRO Land and Water, Glen Osmond, Australia; b Research Center for Eco-Environmental Sciences, Chinese Academy of Sciences, Beijing, China; c CSIRO Computational Informatics, North Ryde, Australia

## Abstract

*Bacillus amyloliquefaciens* strain EBL11 is a bacterium that can promote plant growth by inhibiting the growth of fungi on plant surfaces and providing nutrients as a nonchemical biofertilizer. The estimated genome of this strain is 4.05 Mb in size and harbors 3,683 coding genes (CDSs).

## GENOME ANNOUNCEMENT

A draft genome of *Bacillus amyloliquefaciens* strain EBL11 was obtained by direct sequencing of genomic DNA using Illumina sequencing technology. *B. amyloliquefaciens* is widely known as the source of the BamH1 restriction enzyme and has the ability to produce α-amylase (1). It has also been used as a biological control agent due to its antifungal activity (2). In our previous work, we isolated *Bacillus amyloliquefaciens* EBL11 from rice rhizosphere, and studied its capability of utilizing sweet potato wastewater and promoting plant growth as a biofertilizer (3, 4). We applied these capabilities of strain EBL11 and optimized a clean bioprocess consisting of sweet potato wastewater treatment and plant growth biofertilizer production processes (3). The strain EBL11 is available from the China General Microbiological Culture Collection Centre (CGMCC). The culture identifier is CGMCC No. 6676.

EBL11 was grown on potato-dextrose agar (PDA) medium in a Petri dish. The genomic DNA was prepared by a TruSeq DNA sample prep kit, and sequenced by an Illumina Hiseq sequencer at Beijing Genomic Institute (BGI, Shenzhen, China). The insert size was 500 bp. A total of 4.6 M paired-end reads with length of 90 bp was obtained from sequencing. The raw reads were initially assembled using SOAPdenovo version 1.05. The gaps between contigs and ambiguous patches within contigs were manually filled by aligning individual reads to the contig ends using in-house programs. The genome sequence was annotated by NCBI Prokaryotic Genomes Automatic Annotation Pipeline (PGAAP) (5).

The draft genome of *B. amyloliquefaciens* EBL11 contains 25 contigs with a total length of 4,057,969 bp. The genome comprises 3,683 protein coding genes (CDSs), 16 rRNAs, and 35 tRNAs, with a G+C content of 46.50%. Genome analysis revealed that the genome of *B. amyloliquefaciens* EBL11 has high similarity to strain *B. amyloliquefaciens* FZB42 (identity >96%).

### Nucleotide sequence accession number.

The draft genome sequence of *Bacillus amyloliquefaciens* EBL11 was deposited in DDBJ/EMBL/Genbank under the accession no. JCOC00000000.
